# The Anatomy of Glenoid Concavity—Bony and Osteochondral Assessment of a Stability-Related Parameter

**DOI:** 10.3390/jcm10194316

**Published:** 2021-09-22

**Authors:** Jens Wermers, Michael J. Raschke, Marcel Wilken, Arne Riegel, J. Christoph Katthagen

**Affiliations:** 1 Department of Trauma, Hand, and Reconstructive Surgery, University Hospital Münster, 48149 Münster, Germany; michael.raschke@ukmuenster.de (M.J.R.); christoph.katthagen@ukmuenster.de (J.C.K.); 2 Department of Engineering Physics, University of Applied Sciences Münster, 48565 Steinfurt, Germany; wilken.macl@gmail.com; 3 Department of Radiology, University Hospital Münster, 48149 Münster, Germany; arne.riegel@ukmuenster.de

**Keywords:** glenoid concavity, stability ratio, bony shoulder stability ratio, radiologic assessment, glenoid morphometry, cartilage integrity, glenoid anatomy, osteochondral shoulder stability ratio

## Abstract

Glenoid concavity is a crucial factor for glenohumeral stability. However, the distribution of this stability-related parameter has not been focused on in anatomical studies. In this retrospective study, computed tomography (CT) data and tactile measurements of *n* = 27 human cadaveric glenoids were analyzed with respect to concavity. For this purpose, the bony and osteochondral shoulder stability ratio (BSSR/OSSR) were determined based on the radius and depth of the glenoid shape in eight directions. Various statistical tests were performed for the comparison of directional concavity and analysis of the relationship between superoinferior and anteroposterior concavity. The results proved that glenoid concavity is the least distinctive in anterior, posterior, and anterosuperior direction but increases significantly toward the superior, anteroinferior, and posteroinferior glenoid. The OSSR showed significantly higher concavity than the BSSR for most of the directions considered. Moreover, the anteroposterior concavity is linearly correlated with superoinferior concavity. The nonuniform distribution of concavity indicates directions with higher stability provided by the anatomy. The linear relationship between anteroposterior and superoinferior concavity may motivate future research using magnetic resonance imaging (MRI) data to optimize clinical decision-making toward more personalized treatment of glenoid bone loss.

## 1. Introduction

The glenohumeral morphology enables the shoulder to be the most mobile joint in the human body. The shape of the glenoid socket is relatively flat and small compared to the humeral head, allowing a large range of motion. However, the small bony restraint makes the joint prone to dislocations, injuries, and fractures, especially in young and active males.

Although the anatomical surface of the glenoid appears very flat compared to the humeral head, the glenoid still exhibits some curvature. It has been shown that the osteochondral surfaces are very congruent [1,2]. Furthermore, in the midrange of motion, stability is known to be provided by concavity compression [3]. However, the concavity may differ between patients. Recent finite element methods and biomechanical studies demonstrated that the extent of curvature has a high impact on glenohumeral stability [4,5]. Moroder et al. concluded that the biomechanical effect of a bony defect depends on intraindividual differences in concavity [5]. They thus challenged the current concept of a one- or two-dimensional defect size measurement for decision making in the treatment of bony glenoid defects. Instead, they proposed that the choice of surgical treatment should be optimized in the future by taking the three-dimensional concavity into account. In this way, evaluation of the patient-specific concavity may provide a more precise assessment of glenohumeral stability than is intended with the defect size measurement in the treatment of bony glenoid defects.

The measure of glenoid concavity is not yet well defined. Mathematically, concavity can be expressed as a radius of curvature. For stability analysis, both the radius and the depth of this curvature within a socket are relevant [5]. By now, several ways have been considered to account for concavity. Since the width or depth of a glenoid separately do not capture the curvature of the glenoid, Lazarus et al., used an approximation by calculating the ratio of two times the maximum glenoid depth to the anterior displacement at the point of maximum lateral displacement [6]. Another definition that incorporates concavity is the balance stability angle (BSA), which was defined by Matsen et al. as the maximum angle between the exerted humeral joint reaction force just before the onset of dislocation and the glenoid center line [7,8,9,10].

The most recent definition focusing on concavity derives from Moroder et al., who defined the bony shoulder stability ratio (BSSR) [11]. The BSSR is a mathematical approximation of the stability ratio (SR), which has been used as a measure of stability in many biomechanical and simulative studies [6,12,13,14,15,16,17]. The SR is derived from the maximum dislocating force relative to a joint compression force. When these force distributions are broken down to the glenohumeral morphology, the BSSR is obtained [11]. As recently shown, the BSSR has a high linear correlation with the measured SR [4]. Thus, the BSSR is a stability-related parameter that can clinically be assessed in radiologic data from a measurement of the sphere radius and the glenoid depth [18]. However, it remains unclear if the concavity and BSSR vary around the glenoid. Furthermore, the influence of cartilage on these parameters has not yet been demonstrated.

To date, anatomical studies of the glenohumeral joint have not focused on the extent of concavity. Furthermore, due to a non-spherical shape of the humeral head, concavity can be assumed to have a nonuniform distribution over the glenoid surface [1,19,20]. Dependencies between the superoinferior or anteroinferior shape of concavity can help to further investigate and improve the assessment of this stability-related parameter. In this retrospective study, the anatomy of human cadaveric glenoids was, therefore, analyzed both tangibly and radiologically with a focus on concavity. The objective was to investigate whether concavity varies around the glenoid and how cartilage affects concavity as represented by the BSSR. It was hypothesized that the anatomy of glenoid concavity provides directional stability, which is increased by cartilage, compared to the bony surface.

## 2. Materials and Methods

### 2.1. Specimen Preparation and Data Acquisition

**Figure 1:**
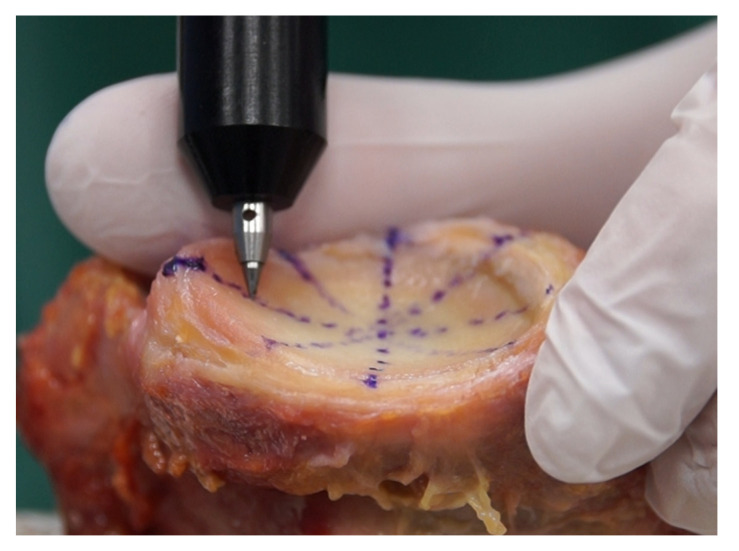
Sampling of the osteochondral glenoid shape with a 3D measuring arm. More than 100 surface points were digitized. In addition, anatomical landmarks were acquired for alignment of the superoinferior and anteroposterior axes on the long and short axes of the glenoid, respectively.

Computed tomography (CT) scans and tactile measurements were performed on *n* = 27 human cadaveric glenoids (12 left, 15 right, 17 female, 10 male, age 79.6 ± 7.4 years). The specimens were thawed overnight and all soft tissue including the capsule, ligaments, muscles, and the labrum were removed. The cartilage was left intact in as good a condition as possible. For this purpose, the labrum was excised by an experienced surgeon to the border between fibrous and homogeneous structures. Specimens with macroscopically visible signs of osteoarthritis, osteophytes, or glenoid bone loss were excluded. The use of specimens for research purposes was approved by IRB (No. 2014-421-f-N, University of Münster, Germany) and the donor bank (University of Lübeck, Germany). CT scan thickness was 0.6 mm, and radiological measurements were performed with Aquarius iNtuition (TeraRecon, Durham, NC, USA) using the multiplanar reconstruction of CT scan data. Tactile measurements of the same specimens were performed using a 3D measuring arm (Absolute Arm 8320-7, Hexagon Metrology, Wetzlar, Germany) by sampling more than 100 points of the osteochondral glenoid surface. The measurement error of the tactile measuring arm was less than 0.05 mm. To avoid a compression of cartilage during tactile measurements, the measurement tip was carefully placed on the surface attempting to avoid deforming contact forces as much as possible. This method is depicted in Figure 1.

### 2.2. Definition of Glenoid Axes and Concavity

**Figure 2:**
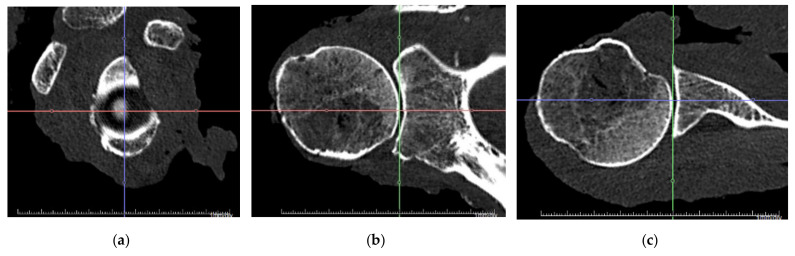
Alignment of joint-specific coordinate system. The superoinferior and anteroposterior axes were aligned with the long and short axes of the glenoid, respectively. The mediolateral axis results orthogonal to the others: (**a**) sagittal view; (**b**) coronal view; (**c**) transversal view.

In both measuring methods, joint-specific coordinate systems were aligned with the long and the short axes of the glenoid. Therefore, the most anterior, posterior, superior, and inferior points on the glenoid rim were digitized. The resulting superoinferior (S/I) and anteroposterior (A/P) axes represented the long and short axes of the glenoid, respectively. The mediolateral axis was obtained by aligning it orthogonally to the other axes. The coordinate system is shown in Figure 2 for CT measurements. This alignment of the coordinate system neglected the effects of physiological retroversion or inclination of the glenoid. In addition, this alignment resulted in a tilt of the coordinate system such that the most anterior and posterior glenoid rims were set at the same mediolateral height as well as the most superior and inferior glenoid rim.

The BSSR was applied as a measure of concavity for both measuring methods. The BSSR is calculated by the following equation:

(1) $$BSSR=\frac{\sqrt{1-{\left(\frac{r-d}{r}\right)}^{2}}}{\frac{r-d}{r}}$$

where (*d*) is the mediolateral depth of the glenoid, determined from the glenoid rim to the deepest point in the cavity, and (*r*) is the radius of a best-fit sphere [11]. However, the *BSSR* in its definition refers only to the bony morphology determined by CT scans. To distinguish this from osteochondral measurements obtained with the 3D measuring arm on the cadaveric specimen, the outcome parameter was renamed to osteochondral shoulder stability ratio (OSSR). The OSSR was calculated with the same equation but using radius (r) and depth (d) measurements while considering the cartilage. Therefore, in this study, the BSSR refers to measurements in CT data, whereas the OSSR refers to measurements on the osteochondral specimen surface.

### 2.3. Measurements and Outcome Parameter

**Figure 3:**
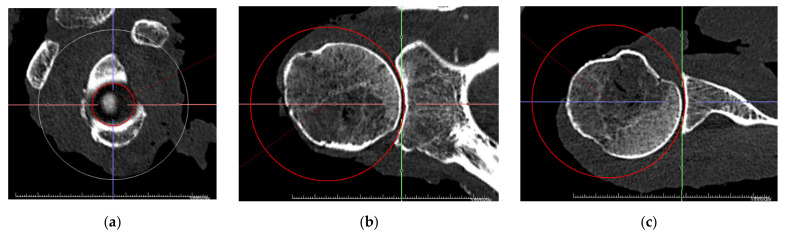
Alignment of a best-fit sphere. The three-dimensional sphere was adjusted to the glenoid surface in a best-fit approach for all three view planes: (**a**) sagittal view; (**b**) coronal view; (**c**) transversal view.

To gain insight into the directional distribution of concavity around the glenoid, the depth (d) was evaluated anterosuperior (AS), anteroposterior (A/P), anteroinferior (AI), superoinferior (S/I), posteroinferior (PI), and posterosuperior (PS). For a right glenoid, these directions correspond to 1:30, 3:00/9:00, 4:30, 6:00/12:00, 7:30, and 10:30 on the clock face. Due to the definition of the coordinate systems, the anterior depth equals the posterior depth, and the superior depth equals the inferior depth, which is the reason why they are summarized as a single direction. The sphere radius was evaluated as the radius of a sphere that best fits the glenoid surface. While this was possible numerically using a minimum mean error approach for the measuring arm data, for the CT data, the sphere was best fitted visually in all three planes using the sphere tool in the radiologic software, as shown in Figure 3.

As neither the humeral head nor the glenoid has a spherical shape [1], the radius (r) was also evaluated as the radius of two-dimensional circles fitted to the glenoid surface in each of the directions considered. For differentiation, this circle radius was termed (r_c_) whereas the sphere radius was denoted as (r_s_). Table 1 summarizes the different methods, outcome parameters, directions, and measurements captured for each specimen. The six directions and calculation of BSSR and OSSR with sphere radius (r_s_) and circle radius (r_c_) resulted in a total of 24 outcome parameters for each specimen.

### 2.4. Statistical Analysis

Outcome parameters were calculated and processed with MATLAB (R2021a, The MathWorks Inc., Natick, MA, USA). Statistics were performed using GraphPad Prism (GraphPad Software Inc., San Diego, CA, USA). The BSSR and OSSR were first calculated based on the circle radius (r_c_) to analyze their glenoid distribution in the directions considered. Repeated measures ANOVA with Šidák’s multiple comparison test were used to compare adjacent directions and to identify significant changes in concavity over the glenoid surface. Furthermore, for each direction separately, BSSR and OSSR outcomes were compared using paired *t*-tests to identify differences between bony and osteochondral concavities. A level of *p* < 0.05 was set for both analyses to identify significance. In a final step, linear regressions were calculated to examine the relationship between superoinferior and anteroposterior concavity. This was carried out using the sphere radius (r_s_) for BSSR and OSSR. The determination coefficient (R^2^) was used to qualify the linearity and predictability of anteroposterior concavity as a function of superoinferior concavity.

## 3. Results

**Figure 4:**
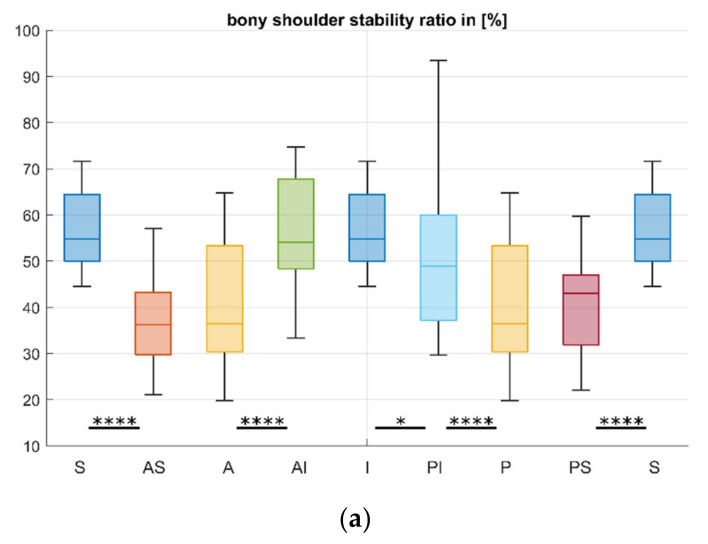

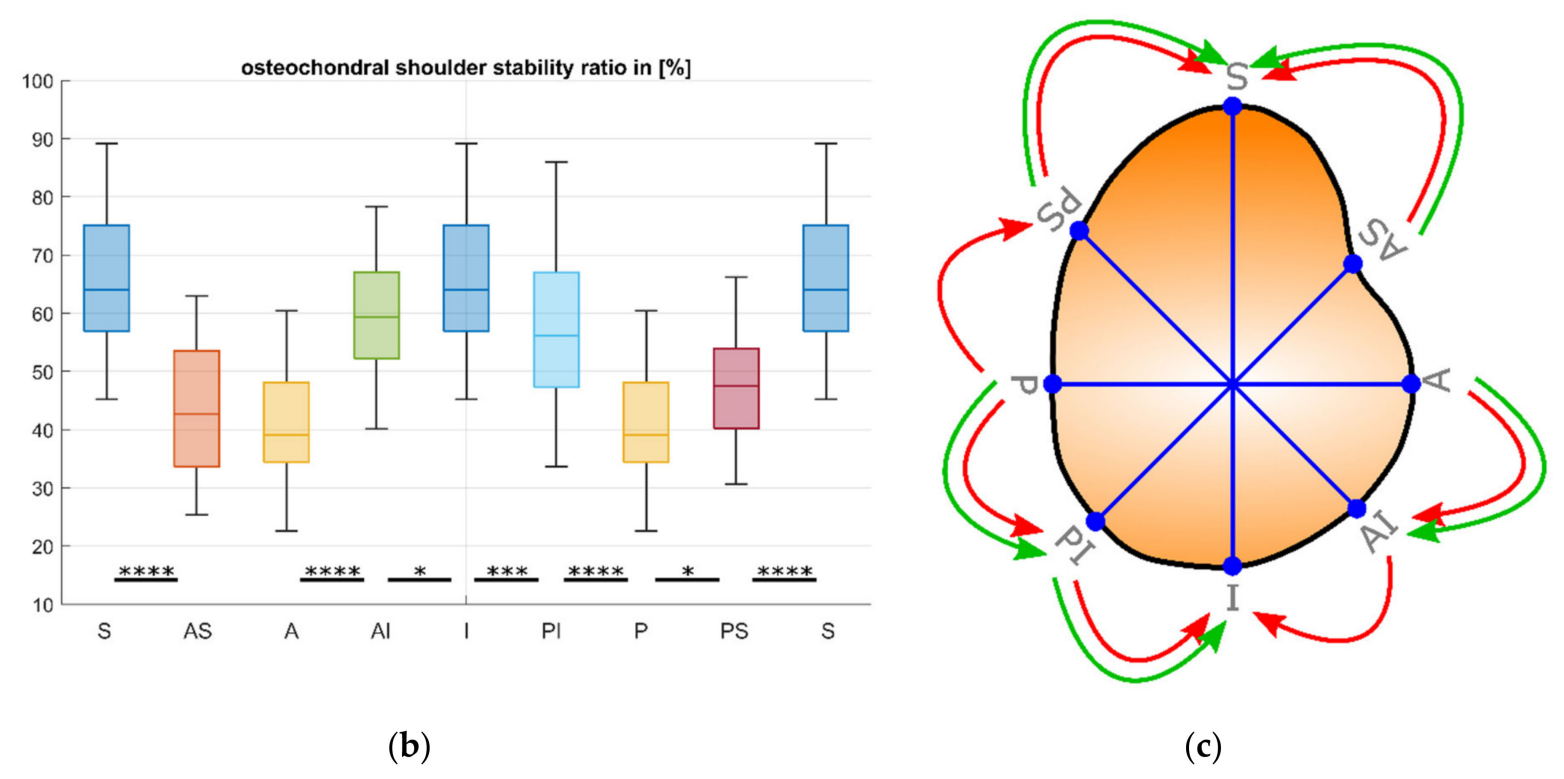
Distribution of concavity for the glenoid directions considered. The BSSR and OSSR increase in superior (S) and inferior (I) directions, whereas the anterior (A), posterior (P), anterosuperior (AS), and posterosuperior (PS) directions provide the least concavity. Note that values of superior and inferior as well as anterior and posterior values remain the same due to definition of the coordinate system: (**a**) BSSR; (**b**) OSSR; (**c**) schematic drawing of glenoid with arrows indicating a significant increase in BSSR (green) and OSSR (red). * *p* < 0.05, *** *p* < 0.001, **** *p* < 0.0001.

The directional analysis of the distribution of BSSR and OSSR is depicted in Figure 4. Statistical analysis revealed a significant increase in concavity when moving from AS and PS to S, from P to PI, as well as from A to AI (each *p* < 0.001) for both outcome parameters.

Furthermore, the BSSR increases significantly from PI to I (*p* = 0.02). However, OSSR additionally showed a significant increase from P to PS (*p* = 0.02), from PI to I (*p* < 0.001) as well as from AI to I (*p* = 0.04). No significant differences were found between AS and A for BSSR and OSSR, and for BSSR measurements between AI and I, as well as between P and PS. The results are presented as mean ± standard deviation in Table 2.

**Figure 5:**
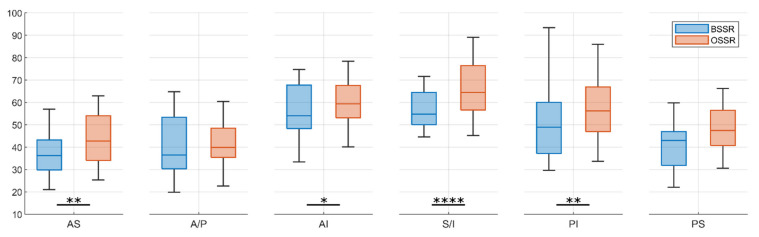
Comparison of BSSR and OSSR for each glenoid direction. The OSSR resulted in higher mean concavities for each direction, however, without a significant effect for anterior (A), posterior (P), and posterosuperior (PS) direction. * *p* < 0.05, ** *p* < 0.01, **** *p* < 0.0001.

Comparison of BSSR and OSSR for each direction separately revealed significantly higher concavity for the OSSR in AS (*p* < 0.01), AI (*p* = 0.03), S and I (*p* < 0.001), as well as PI (*p* < 0.01) direction. For the other directions, the mean values of BSSR measurements were also lower than the OSSR measurements but without a statistical significance. The results are shown in Figure 5.

**Figure 6:**
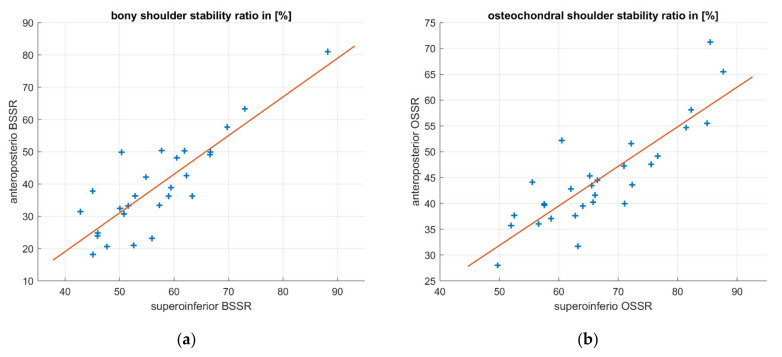
Linear regression of anteroposterior concavity as a function of superoinferior concavity for BSSR and OSSR separately. Both measurement methods reveal a high linear correlation that indicates predictability of anteroposterior concavity only by measuring superoinferior concavity: (**a**) BSSR; (**b**) OSSR.

The relationship between anteroposterior (A/P) and superoinferior (S/I) concavity was examined using linear regressions. The resulting linear relationships and raw measurements are shown in Figure 6. The determination coefficient resulted in R^2^ = 0.72 for both regression models indicating a high linear correlation. The linear regressions resulted in the following equations:

(2) $$BSSR_{A/P}=-28.12+1.2\times BSSR_{S/I}$$

(3) $$OSSR_{A/P}=-6.45+0.77\times OSSR_{S/I}$$

which represent an approximation of anteroposterior concavity as a function of superoinferior concavity.

## 4. Discussion

The results of this study allow interpretation of the following findings: (1) glenoid concavity is nonuniformly distributed around the anatomic surface but increases in the superior and inferior extent of the glenoid; (2) the osteochondral concavity is more pronounced than its bony representation; (3) for both approaches, the anteroposterior concavity is linearly correlated with the superoinferior concavity.

The anatomy of glenoid concavity is a crucial factor contributing to glenohumeral stability. Loss of concavity, which was recently mathematically represented by the BSSR, is directly and linearly related to the loss of SR, a widely used biomechanical estimate for glenohumeral stability [4,5]. A deeper knowledge of the bony and osteochondral concavity distribution is essential to understand the important stability effect of the anatomy. However, glenoid concavity has not yet been focused on in anatomical studies. In this study, radiologic and tactile measurements of human cadaveric specimens were analyzed with respect to the anatomy of glenoid concavity.

The results of this analysis proved a nonuniform distribution of concavity over the glenoid surface for both, the bony (BSSR) and osteochondral (OSSR) representation. While a significant increase was found toward the superior and inferior glenoid, the least differences were detected between the anterior and anterosuperior concavity. Furthermore, the bony concavity differed insignificantly between the posterosuperior and posterior glenoid. Based on the relation of concavity to the SR, these findings indicate lower bony and osteochondral stability that is provided by the anatomical shape of the glenoid, particularly in the anterior-/anterosuperior and posterior-/posterosuperior directions.

The large differences in directional concavity suggest that the three-dimensional shape of the glenoid has a major influence on the stability of the joint. In particular, the inferior direction with the greatest concavity is the least affected by bony defects. In contrast, the lack of concavity in the anterior direction may be a reason why the majority of dislocations occur anteriorly and why anterior bone loss occurs most frequently perpendicular to the 3 o’clock direction [21,22]. Furthermore, the determined values of BSSR and OSSR ranged from below 20% to more than 80 %, thus confirming large intraindividual constitutional differences in the glenoid shape. Consequently, the results support the concept proposed by Moroder et al. that the three-dimensional anatomy of glenoid concavity is more relevant than the one- or two-dimensional assessment of a bony defect and should be considered in the future treatment of bony defects [5].

Two different methods were used to assess concavity in this study. While the BSSR was determined using CT scans, the OSSR was acquired based on tactile measurements of the osteochondral surface. The consideration of cartilage led to a higher mean concavity in the OSSR compared to its bony counterpart on average for all directions, for most in a significant manner. Cartilage provides a more congruent surface to the humeral head but may also add concavity, especially in the superoinferior, anterosuperior, posteroinferior, and anteroinferior regions. These findings fit with the nonuniform cartilage distribution, which is particularly pronounced by the bare spot [1,2,23].

Another finding of this study is a high linear correlation between the superoinferior and anteroposterior concavity. Incorporating concavity into clinical decision making in the treatment of bony glenoid defects may help to improve surgical outcomes and tailor the treatment to a more personalized approach. In the case of traumatic glenoid bone loss, the initial anteroposterior concavity may be of great interest. However, many patients suffering from glenoid bone loss do not present with preexisting radiologic data of the intact glenoid. A few studies have proposed formulas to estimate native glenoid width based on the length [24,25]. However, it has been shown that the width of bony defects has inaccurate information about stability [4]. The approximation of initial anteroposterior concavity by measuring only the superoinferior concavity in terms of BSSR or OSSR may be a more precise approach to manage the loss of bony glenoid. As demonstrated in the results, anteroposterior concavity can be calculated as a function of superoinferior concavity. The linear coefficients in Equations (2) and (3) are quite different, indicating a high impact of cartilage on concavity distribution. However, due to a small sample size of n = 27, future studies must be carried out to identify an accurate factor or equation for this assessment.

There are several limitations in this study. The outcome parameters were identified by entirely different measuring methods. The enhancement of concavity by cartilage could be obscured by imprecise measurements in CT data, which has an inaccuracy of more than 10 times greater than the measuring arm. Furthermore, the transition between cartilage and labrum is not always precisely distinguishable, and tactile measurements of the cartilage surface may have resulted in small, unavoidable compressions of the cartilage. This leads to greater inaccuracies and deviations than indicated by the technical accuracy of the measuring arm. However, the main dependencies found in this study were identified independently in both measuring methods. This motivates further research to assess concavity in radiologic data such as magnetic resonance imaging (MRI) data. Other limitations are the high average age of specimens and the relatively low sample size. Further research should be carried out to analyze differences in age, sex, as well as intraindividual differences in contralateral glenoids. Furthermore, the results of tactile measurements may not be directly applicable in clinical practice, as they are not reproducible by radiologic measurements. Nevertheless, the results of this study provide important insights that suggest further investigation on the anatomy of glenoid concavity in MRI data, considering cartilage and labrum. In addition, the results on concavity distribution and its representation by the BSSR and OSSR may stimulate further research toward more personalized treatment approaches.

## 5. Conclusions

The nonuniform distribution of concavity indicates directions with higher stability provided by the anatomy. The linear relationship between anteroposterior and superoinferior concavity may motivate future research using magnetic resonance imaging (MRI) data to optimize clinical decision making toward more personalized treatment of glenoid bone loss.

## Figures and Tables

**Table 1:** Summary of methods, directions, measurements, and outcome parameters.

Method	Directions	Measurements	Outcome Parameter
CT scan	Anterosuperior (AS)	Depth (d)	BSSR
Measuring arm	Anterior/posterior (A/P)	Sphere radius (r_s_)	OSSR
	Anteroinferior (AI)	Circle radius (r_c_)	
	Superior/inferior (S/I)		
	Posteroinferior (PI)		
	Posterosuperior (PS)		

**Table 2:** Results of BSSR and OSSR in % for glenoid directions considered. Results are presented as mean ± standard deviation.

Direction	BSSR in [%]	OSSR in [%]
Anterosuperior (AS)	37.1 ± 15.0	42.9 ± 11.1
Anterior/posterior (A/P)	40.6 ± 16.2	41.0 ± 11.0
Anteroinferior (AI)	56.6 ± 16.3	61.1 ± 13.1
Superior/inferior (S/I)	57.0 ± 10.3	65.9 ± 11.9
Posteroinferior (PI)	51.0 ± 15.9	57.7 ± 14.1
Posterosuperior (PS)	42.4 ± 13.2	47.4 ± 10.3

## Data Availability

Data is contained within the Supplementary Material.

## Supplementary Materials

The following are available online at https://www.mdpi.com/article/10.3390/jcm10194316/s1, Supplementary Material 1: supplementary material table.
